# Screening for cancer in unprovoked venous thromboembolism

**DOI:** 10.1097/HS9.0000000000000205

**Published:** 2019-06-30

**Authors:** Salma Shivji, Marc Carrier

**Affiliations:** 1 Division of Hematology and Hematological Malignancies, Departments of Medicine and Community Health Sciences, University of Calgary, Calgary, Canada; 2 Department of Medicine, Ottawa Hospital Research Institute, University of Ottawa, Ontario, Canada

**Take home messages**

- The incidence of occult malignancy in patients with unprovoked venous thromboembolism (VTE) is up to 5% and therefore VTE can be the first manifestation of cancer.
- Strategies that add extensive imaging to routine investigations including age and sex-appropriate screening have not resulted in a significant increase in occult cancer diagnosis or improvement of patient outcomes.
- Clinical scores have been developed which look to target screening techniques toward patients at highest risk of malignancy, but many have not yet been validated for clinical practice.

## Introduction

**Figure 1:**
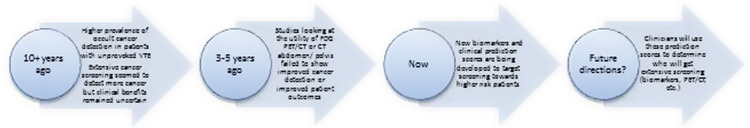
**Timeline of cancer screening in unprovoked venous thromboembolism**.

Venous thromboembolism (VTE) can be the first indicator of an underlying cancer. Older studies have indicated that in the 12 months following an unprovoked VTE event, up to 10% of patients are diagnosed with cancer.^1▪^ However, more recent multicenter, open-label, randomized studies have detected a much lower rate of cancer diagnosis in these patients. In the SOME trial conducted in Canada,^2▪^ and in the MVTEP trial conducted in France,^3▪^ only 4.5% and 5.6% of patients with unprovoked VTE were later found to have a cancer diagnosis. Similarly, a recently conducted systematic review and individual patient data meta-analysis (IPDMA) of 10 studies reported a 1-year cancer diagnosis rate of 5.2% (95% confidence interval [CI]: 4.1–6.5).^4▪^ Information about the incidence of cancer diagnosis beyond the first year of follow up comes from the MVTEP study and the IPDMA.^3▪^^,^^4▪^ Patients from those studies had a cancer diagnosis rate of 1% and 1.1% in the second year of follow up. Regardless of the prevalence of cancer in these patients, clinicians often feel compelled to look for an occult cancer in an attempt to better patient outcomes (Fig. 1).

## Current state of the art

Given that a VTE presentation may be the first presentation of a patient having an occult cancer, there have been many studies looking at whether an extensive or limited approach is better for cancer screening in these patients. Generally, limited screening consists of a complete history and physical examination, routine bloodwork, chest radiography as well as age- and gender-appropriate screening and a more extensive screening includes additional diagnostic imaging (eg, computed tomography [CT], ultrasonography). Intuitively, if more cancers can be discovered at an earlier stage as a result of more extensive screening, then it should lead to improved patient outcomes. However, many studies have failed to show that extensive occult cancer screening leads to a greater cancer diagnostic rate or the detection of earlier stage tumors. The SOME trial which randomized 854 patients to a limited or a more extensive screening strategy by adding a CT of the abdomen and pelvis, reported no difference in missed cancer diagnosis between the 2 groups. There was also no statistically significant increase in occult cancer diagnosis in the 1-year follow-up period from 3.2% (14 out of 431 patients) to 4.5% (19 out of 423 patients) in the limited and extensive screening groups, respectively (*P* = 0.28).^2▪^ Furthermore, the study could not detect a statistically significant difference in the mean time to cancer diagnosis nor in cancer-related mortality between the 2 groups.^2▪^ Similarly, the recent IPDMA data, despite showing that an extensive cancer screening method yielded a twofold higher probability of occult cancer detection, also did not show any effect on the detection of early cancers, nor any difference in overall or cancer-related mortality.^4▪^

There have been studies looking at whether a more sensitive diagnostic modality, such as 18F-fluorodeoxyglucose positron emission tomography (FDG PET)/CT to detect earlier cancers, is effective. FDG PET/CT is routinely utilized for the diagnosis, staging, and restaging of various cancers. The MVTEP trial could not find a statistically significant difference in the rate of cancer diagnosis when looking at a limited screening strategy compared to the addition of FDG PET/CT. Occult cancers were detected in 5.6% and 2.0% of patients in the FDG PET/CT and limited screening groups, respectively (absolute risk difference 3.6%, 95% CI −0.4 to 7.9; *P* = 0.07).^3▪^ Cancers in early stages were detected in 64% of patients (7/11) in the FDG PET/CT group compared with in 50% of patients (2/4) in the limited screening group (*P* = 1.00).^3▪^

An additional study looked at the healthcare-related costs of FDG PET/CT in a public healthcare setting and could not show a clear benefit of the addition of a PET screening strategy to current age and gender appropriate screening techniques. The cost of the additional FDG PET/CT was C$26,840.19 or €15,370.45 per one avoided cancer diagnosis and C$3412.85 or €2162.83 per quality adjusted life year gained in that analysis.^5^

## Future perspective

Although the prevalence of cancer in patients affected by VTE may not be as high as previously thought, the patient populations studied were quite heterogeneous. Recent research has focused on determining if there is a subset of patients who experience their first unprovoked VTE that may be at higher risk of occult cancer detection. The post hoc data from the SOME trial found that persons of age ≥60 years, with previous provoked VTE, and current smoker status might predict occult cancer in this population.^6^ The MVTEP trial shows that patient characteristics such as being male and age as well as having a high leukocyte or platelet count may be associated with greater occult cancer detection.^7^ The IPDMA results found that age was the most important predictor of occult cancer detection, and surprisingly found that gender, smoking status, and previous VTE were not as predictive.^4▪^

Investigators of the RIETE study developed and validated a clinical prediction rule for the risk of occult cancer in VTE.^4▪^ The score looks at 7 items: male gender; age > 70 years; chronic lung disease; anemia; elevated platelets; and recent surgery. A score of ≤ 2 was associated with a 5.8% and 3.6% risk of occult cancer in the original and the MVTEP validation cohort, respectively; while a score of ≥3 was associated with a 12% and 11.8% risk of occult cancer in the original and validation cohorts, respectively.^4▪^^,^^8▪^ The use of this score in clinical practice has not yet been adopted into recent guidelines but is an area of much interest to clinicians.

The association between cancer and unprovoked VTE is a topic that has generated much research in recent years. It is commonly accepted that an extensive search for an occult malignancy is not necessary, but select patients still have an increased risk of an underlying cancer. Many questions remain such as how best to choose which patients to screen in order to improve patient outcomes.
